# Immunosuppressive activity enhances central carbon metabolism and bioenergetics in myeloid-derived suppressor cells *in vitro* models

**DOI:** 10.1186/1471-2121-13-18

**Published:** 2012-07-04

**Authors:** Ines Hammami, Jingkui Chen, Frederic Murschel, Vincenzo Bronte, Gregory De Crescenzo, Mario Jolicoeur

**Affiliations:** 1 Department of Chemical Engineering, Ecole Polytechnique de Montréal, 2500 Chemin de Polytechnique, H3T-1J4, Montreal, QC, Canada; 2 Department of Pathology, Immunology Section, Verona University, P. le L.A. Scuro, 10 – 37134, Verona, Italy

**Keywords:** Myeloid-derived suppressor cells, GM-CSF, IL-6, MSC-1 cells, Central carbon metabolism, Bioenergetics

## Abstract

**Background:**

The tumor microenvironment contains a vast array of pro- and anti-inflammatory cytokines that alter myelopoiesis and lead to the maturation of immunosuppressive cells known as myeloid-derived suppressor cells (MDSCs). Incubating bone marrow (BM) precursors with a combination of granulocyte-macrophage colony-stimulating factor (GM-CSF) and interleukin-6 (IL-6) generated a tumor-infiltrating MDSC-like population that impaired anti-tumor specific T-cell functions. This in vitro experimental approach was used to simulate MDSC maturation, and the cellular metabolic response was then monitored. A complementary experimental model that inhibited L-arginine (L-Arg) metabolizing enzymes in MSC-1 cells, an immortalized cell line derived from primary MDSCs, was used to study the metabolic events related to immunosuppression.

**Results:**

Exposure of BM cells to GM-CSF and IL-6 activated, within 24 h, L-Arg metabolizing enzymes which are responsible for the MDSCs immunosuppressive potential. This was accompanied by an increased uptake of L-glutamine (L-Gln) and glucose, the latter being metabolized by anaerobic glycolysis. The up-regulation of nutrient uptake lead to the accumulation of TCA cycle intermediates and lactate as well as the endogenous synthesis of L-Arg and the production of energy-rich nucleotides. Moreover, inhibition of L-Arg metabolism in MSC-1 cells down-regulated central carbon metabolism activity, including glycolysis, glutaminolysis and TCA cycle activity, and led to a deterioration of cell bioenergetic status. The simultaneous increase of cell specific concentrations of ATP and a decrease in ATP-to-ADP ratio in BM-derived MDSCs suggested cells were metabolically active during maturation. Moreover, AMP-activated protein kinase (AMPK) was activated during MDSC maturation in GM-CSF and IL-6–treated cultures, as revealed by the continuous increase of AMP-to-ATP ratios and the phosphorylation of AMPK. Likewise, AMPK activity was decreased in MSC-1 cells when L-Arg metabolizing enzymes were inhibited. Finally, inhibition of AMPK activity by the specific inhibitor Compound C (Comp-C) resulted in the inhibition of L-Arg metabolizing enzyme activity and abolished MDSCs immunosuppressive activity.

**Conclusions:**

We anticipate that the inhibition of AMPK and the control of metabolic fluxes may be considered as a novel therapeutic target for the recovery of the immunosurveillance process in cancer-bearing hosts.

## Background

Tumor growth and progression are critically controlled by alterations in the microenvironment, often caused by aberrant expression of tumor-derived soluble factors (TDSFs) [1]. In addition to stimulating tumor cell proliferation, stromal activation and angiogenesis, TDSFs promote the maturation/recruitment of myeloid-derived suppressor cells (MDSCs) that inhibit the tumor-specific functions of CD8^+^ and CD4^+^ T lymphocytes [2]. MDSCs have been characterized in tumor-bearing mice by the expression of the surface markers CD11b and Gr-1 [3]. Their immunosuppressive functions depend on two enzymes: i) inducible nitric oxide synthase (iNOS) that converts L-arginine (L-Arg) to nitric oxide (NO) and L-citrulline, and ii) arginase 1 (ARG1) that metabolizes L-Arg into urea and L-ornithine. A decreased availability of L-Arg combined with the accumulation of NO derivatives (NO_2_^-^, NO_3_^-^, N_2_O_3_) trigger the inhibition of T-cell function and proliferation, and induce cell death [3].

Despite considerable progress in understanding MDSC maturation both *in vivo* and in vitro, mechanisms of immunosuppression at the metabolic level are still unclear. Specific immune functions, including antigen processing and presentation, cytokinesis and activation, are known to be energetically-costly [4]. Moreover, immune cells, such as lymphocytes and macrophages, modulate their metabolism and respiration to fulfill their energy requirements. The energy metabolism of immune effector cells was thus considered a potential target for immunotherapy. Indeed, drugs that affect cell bioenergetics can reduce ATP production, for the treatment of psoriasis, rheumatoid arthritis, and cardiac arrhythmia. These drugs act either by inhibiting reactions related to substrate oxidation or by increasing proton permeability through the mitochondria, in turn resulting in the uncoupling of oxidative phosphorylation [4].

Therefore, to better understand the nutritional and energy requirements of MDSCs, two complementary experimental models were used. The first consisted of in vitro maturation of bone marrow (BM)-derived MDSCs using a combination of granulocyte-macrophage colony-stimulating factor (GM-CSF) and interleukin (IL)-6, as previously reported [5], which generated a CD11b^+^/Gr-1^low^ population, the most tolerogenic and immunosuppressive sub-population among CD11b^+^/Gr-1^+^ cells [6]. This cell population expresses iNOS and ARG1, and has a similar genetic signature to tumor-infiltrating MDSCs [5]. To discern whether the metabolic changes that occur during maturation are related to the direct effects of GM-CSF and IL-6 on metabolic pathways and nutrient transporters, or are associated with the activation of iNOS and ARG1, we used a second model that inhibited iNOS and ARG1 activity in MSC-1 cells, an immortalized cell line derived from mouse MDSCs. Being phenotypically similar to primary MDSCs, the MSC-1 cell line represents a relevant model system. MSC-1 cells constitutively expressed iNOS and ARG1 and inhibited antigen-specific proliferation and functions of cytotoxic T-lymphocytes without any additional treatment with specific cytokines or endotoxins, as previously demonstrated [7,8]. Comparing the results, we further confirmed the MSC-1 cell line as a model system.

iNOS and ARG1 activities were respectively inhibited by *N*-[3-(aminomethyl)-benzyl]-acetamidine (1400 W) and [(S)-(2-boronoethyl)-L-cysteine] (BEC). 1400 W was selected as it is a tight-binding and highly selective inhibitor of iNOS exhibiting the highest potency of reported iNOS inhibitors [9]. BEC is a potent slow-binding competitive and selective inhibitor of ARG1 [10]. Both 1400 W and BEC inhibitors act directly on enzymatic activity, without affecting enzyme mRNA or protein levels [9,10].

Results of the present study suggest MDSC maturation and immunosuppressive potential are accompanied by an increase in the central carbon metabolism activity level and bioenergetic status. This study also emphasizes the key role of AMPK in the maintenance of MDSCs immunosuppressive activity.

## Results

### Immunosuppressive activity of BM-derived MDSCs and MSC-1 cells

**Figure 1:**
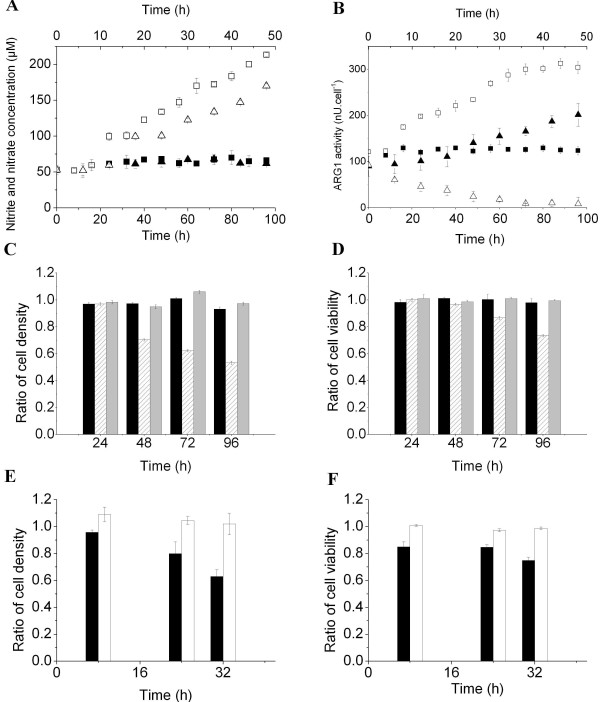
** Immunosuppressive activity of BM-derived MDSCs and MSC-1 cells.** BM cells were extracted from 6 to 8-week-old C57BL/6 mice and cultured for 4 days in the presence of GM-CSF and IL-6 (40 ng/ml each, except for the control cultures). MSC-1 cells were cultured for 48 h in the presence of 100 μM of 1400 W and 5 μM of BEC (except for control cultures). (**A**) Nitrite and nitrate concentration based on the Griess reaction. (**B**) ARG1 activity: one unit (U) of ARG1 activity is defined as the enzyme activity that catalyses the production of 1 μmol urea/min. Filled and empty symbols (■, □) correspond to BM cells and BM-derived MDSC cultures, respectively. Filled and empty symbols (▴, ▵) correspond to MSC-1 cells and 1400 W and BEC-treated MSC-1 cells, respectively. The same nomenclature is used for all figures unless specified. (**C, D**) Ratios (referenced to the control culture) of Jurkat cell density and viability, respectively. Jurkat cells were cultured for 24 h in the presence of untreated BM cells (Black), BM cells exposed to GM-CSF and IL-6 for 24, 48, 72 or 96 h (hatched) and cytokines (Grey). (**E, F**) Ratios (referenced to the control culture) of Jurkat cell density and viability, respectively. Jurkat cells were inoculated in the presence of MSC-1 cells (Black) or MSC-1 cells pre-cultured for 12 h in the presence of 1400 W (100 μM) and BEC (5 μM) (White). Ratios are based on the cell density and viability of the control culture, which consists of Jurkat cells inoculated in inserts (500 μl at 0.2 × 10^6^ cells/ml) placed in wells containing 500 μl of complemented culture medium. The same control culture is considered for subsequent cytotoxicity tests.

The enzymatic activities of iNOS and ARG1 were extremely low in BM cell culture, as revealed by a constant concentration of nitrate and nitrite in the supernatant (62.86 ± 4.25 μM) and constant ARG1 activity at 124.93 ± 3.48 nU/cell throughout the culture (Figure 1A, B). Thus, although CD11b^+^/Gr-1^+^ cells can be detected at sizeable number in BM of healthy mice [3], BM cells have no immunosuppressive potential (Figure 1C, D). In fact, a cytotoxicity assay showed that BM cells did not affect Jurkat cell growth or viability (Figure 1C, D). In contrast, treating BM cells with GM-CSF and IL-6 resulted in a marked increase of ARG1 and iNOS activity after 16 h and 24 h, respectively. Nitrate and nitrite continuously accumulated at a rate of 6.64 ± 0.09 fmol/cell/h while ARG1 activity shifted from 122.17 ± 0.65 nU/cell at inoculation to 174.79 ± 3.58 nU/cell at 16 h, and continuously increased until a quasi-stable level of 304.30 ± 4.11 nU/cell was attained at 72 h. The accumulation of nitrate and nitrite suggested that L-Arg was permanently present in the supernatant, otherwise iNOS and ARG1 activities would be down-regulated and a loss of BM-derived MDSC immunosuppressive activity would be observed. The ability of BM-derived MDSCs to inhibit Jurkat cell growth and decrease their viability was detected after 48 and 72 h of treatment, respectively (Figure 1C, D). This delay was likely due to an increase in the proportion of MDSCs in the cytokine-treated BM cell suspension, since BM cells were not synchronized prior to GM-CSF and IL-6 addition. More specifically, after 96 h of treatment, BM-derived MDSCs decreased Jurkat cell density and viability by 46.6 ± 1.07 and 26.47 ± 0.89%, respectively. Culturing Jurkat cells in the presence of GM-CSF and IL-6 did not affect cell growth or viability (Figure 1C, D), suggesting that the effects observed in the treated-BM and Jurkat mixed cell culture were caused by the maturation of BM-derived MDSCs, and not to cytokine-related cytotoxic effects.

It is important to note that the phenotype and genomic profile of GM-CSF and IL-6-treated BM cells were not re-verified herein, since we used the same protocol as described by Marigo et al., including mice species, age, sex, euthanasia protocol, BM cell extraction, and chemical products for cell extraction and culture [5]. As discussed, the L-Arg metabolizing enzymes, iNOS and ARG1, were both activated following GM-CSF and IL-6 exposure, and the resulting cells were immunosuppressive. This suggests that cytokine-treated BM cells behave as MDSCs at the immunosuppression level.

However, culturing MSC-1 cells in the presence of 1400 W and BEC triggered the rapid inhibition of iNOS activity, since the concentration of nitrate and nitrite, used as a marker of iNOS activity, remained constant at 50.03 ± 4.57 μM, whereas nitrate and nitrite accumulated at a rate of 2.33 ± 0.09 μM/h in the control culture (Figure 1A). The high concentrations of nitrate and nitrite in the control culture did not affect MSC-1 cell viability, which was maintained at higher than 97% throughout the 48 h culture (data not shown). The inhibition of ARG1 activity occurred at different rates (Figure 1B). ARG1 activity was decreased by 50% during the first 12 h of treatment, and then decreased from 45.78 ± 9.78 nU/cell/h at 12 h, to 9.42 ± 0.64 nU/cell/h at 36 h, and remained relatively stable until the end of the culture (48 h).

High concentrations of inhibitors (100 μM for 1400 W and 5 μM for BEC) in comparison with the 1400 W half maximal effective concentration (EC_50_) of 0.8 ± 0.3 μM [9] and the BEC dissociation constant (*K*_*i*_) of 0.5 ± 0.1 μM [11]), were used to achieve maximum inhibition of iNOS and ARG1 activity in newly divided MSC-1 cells. These concentrations did not induce loss of cell viability (data not shown) or growth inhibition, and similar specific growth rates were observed for the control culture (0.047 ± 0.001 per h) and cells treated with 1400 W and BEC (0.046 ± 0.003 per h). Moreover, the use of high concentrations of 1400 W and BEC are commonly reported in the literature without any noticeable cytotoxic effect on several different cell types [12-15].

The cytotoxicity assay showed that co-culturing Jurkat and MSC-1 cells pre-treated with 1400 W and BEC reduced the immunosuppressive potential of MSC-1 cells. Indeed, Jurkat cell density (Figure 1E) and viability (Figure 1F) were similar to those measured in the control culture (Jurkat cells only). In contrast, in the presence of untreated MSC-1 cells, Jurkat cell density and viability after 32 h were decreased by 37.08 ± 5.03% and 25.25 ± 2.32%, respectively, when compared to the control culture (*p* < 0.05). Exposure of Jurkat cells to 1400 W and BEC in the absence of MSC-1 cells did not affect their growth or viability (data not shown).

### Immunosuppression activity affects the nutritional profile of BM-derived MDSCs and MSC-1 cells

**Figure 2:**
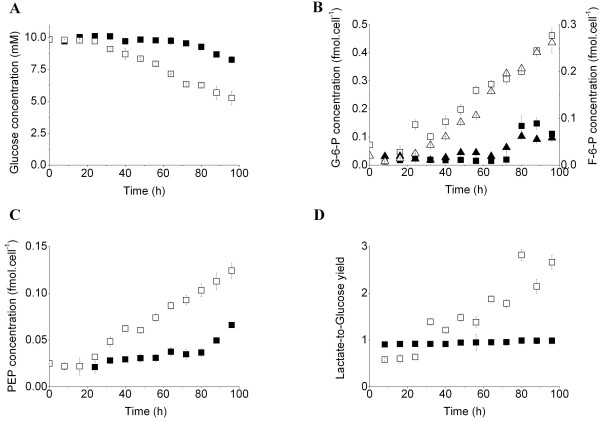
** GM-CSF and IL-6 modulate BM-derived MDSC glucose metabolism.** (**A**) Glucose concentration in the supernatant. (**B**) Cell specific concentrations in G-6-P (squares) and F-6-P (triangles). (**C**) Cell specific PEP concentration. (**D**) Lactate-to-glucose yield. BM cells and BM-derived MDSCs were extracted using cold methanol and organic acids were analyzed using a UPLC-MS/MS system.

BM-derived MDSCs exhibited a low specific growth rate of 0.13 ± 0.02 per d from 32 h, whereas no cell growth was detected in the untreated BM cell culture (control culture) although cells stayed viable throughout the culture. BM-derived MDSCs consumed glucose at a high rate of 0.243 ± 0.008 pmol/cell/h from 24 h, whereas the control culture scarcely consumed glucose during the first 72 h and then at a rate of 0.278 ± 0.013 pmol/cell/h (Figure 2A). The increase of glucose uptake in the BM-derived MDSC culture was accompanied by the accumulation of glycolysis intermediates from 32 h. The cell specific concentration of glucose-6-phosphate (G-6-P) and fructose-6-phosphate (F-6-P) increased at rates of 5.173 ± 0.084 and 3.573 ± 0.005 × 10^-3^ fmol/cell/h, respectively (Figure 2B), showing that G-6-P is mostly processed through glycolysis and lower quantities enter the pentose phosphate pathway (PPP). The increase of cell specific concentrations of glycolysis intermediates in the BM cell culture, coincided with the increased glucose consumption rate (72 h) (Figure 2B, C). Moreover, the enhanced glucose uptake was accompanied by decreased cell respiration. The specific oxygen consumption rate decreased from 103.42 ± 6.14 fmol/cell/h at 24 h to 41.88 ± 3.62 fmol/cell/h at 96 h (data not shown). The lactate-to-glucose yield, in the GM-CSF and IL-6 -treated BM cell culture, was relatively stable at 0.61 ± 0.02 for the first 24 h and then rapidly increased to 1.39 ± 0.04 at 32 h and reached values higher than 2.00 after 80 h (Figure 2D), suggesting that another source of carbon contributed to the accumulation of lactate. Conversely, the lactate-to-glucose yield of the BM cell culture was almost stable at 0.95 ± 0.02 (Figure 2D).

**Figure 3:**
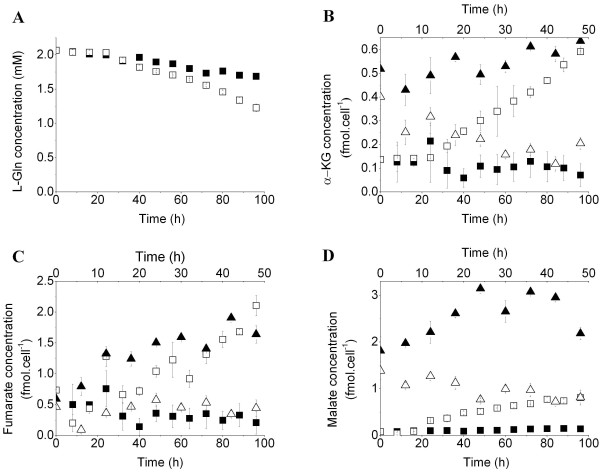
** Regulation of glutaminolysis and TCA cycle during immunosuppression.** (**A**) L-Gln concentration in supernatant. (**B, C, D**) Specific cell concentrations of α-KG, fumarate and malate, respectively.

The activation of iNOS and ARG1, within the first 24 h, was followed by changes in glycolytic intermediates and cell respiration levels at 32 h. We then investigated whether iNOS and ARG1 activation has a direct effect on the metabolic shifts. The characterization of MSC-1 and 1400 W and BEC-inhibited MSC-1 cell nutritional profiles led to similar interpretations. The inhibition of iNOS and ARG1 activities resulted in the decrease of specific glucose consumption rate from 0.292 ± 0.018 pmol/cell/h (control culture) to 0.153 ± 0.008 pmol/cell/h. This was accompanied by an increase in cell specific oxygen consumption rate from 0.066 ± 0.005 pmol/cell/h (control culture) to 0.108 ± 0.001 pmol/cell/h. The ratio of the consumption rates of oxygen-to-glucose increased from 0.23 ± 0.03 in the control culture to 0.72 ± 0.5 in the 1400 W and BEC-treated cultures. In agreement with the lower glucose uptake rate, lactate production rate was also reduced from 0.511 ± 0.028 pmol/cell/h (control culture) to 0.292 ± 0.021 pmol/cell/h when iNOS and ARG1 were inhibited. However, the lactate-to-glucose yield slightly increased from 1.78 ± 0.18 in the control culture to 1.92 ± 0.24 in the 1400 W and BEC-treated culture. Furthermore, BM-derived MDSCs consumed L-Gln at a higher rate (0.040 ± 0.002 pmol/cell/h) than that observed in the control culture (0.021 ± 0.005 pmol/cell/h) (Figure 3A). This was confirmed by a decrease in the L-Gln consumption rate from 0.086 ± 0.004 pmol/cell/h (control culture) to 0.052 ± 0.001 pmol/cell/h in the presence of 1400 W and BEC. Interestingly, only slight differences in nutrient consumption and metabolite production rates were observed between MSC-1 cells and BM-derived MDSCs cultures, and similar results have been observed for both models in the current study.

The stimulation of glycolysis and glutaminolysis in BM-derived MDSC cultures resulted in an increase in the cell content of TCA cycle intermediates. Particularly, α-ketoglutarate (α-KG), which can be derived from isocitrate or L-Gln, accumulated at a rate of 5.873 ± 0.005 × 10^-3^ fmol/cell/h (Figure 3B). Similarly, the cell specific concentration of fumarate, a by-product of the conversion of argininosuccinate into L-Arg, increased at a rate of 20.378 ± 0.005 × 10^-3^ fmol/cell/h after 24 h (Figure 3C), strongly suggesting that maturation of BM cells to MDSCs was accompanied by the activation of the L-Arg recycling pathways. Moreover, measurement of the cell specific concentrations of both sources of pyruvate, malate, which is derived from fumarate, and phosphoenolpyruvate (PEP), indicated that malate was 5-fold more concentrated than PEP (Figures 2C, 3D). Consistent with the down-regulation of the net uptake rates of major nutrients (glucose and L-Gln), inhibiting iNOS and ARG1 activities resulted in a decrease of the specific cell concentrations of TCA cycle substrates, particularly α-ketoglutarate, fumarate and malate (Figure 3B, C, D).

### Immunosuppression-related bioenergetics

**Figure 4:**
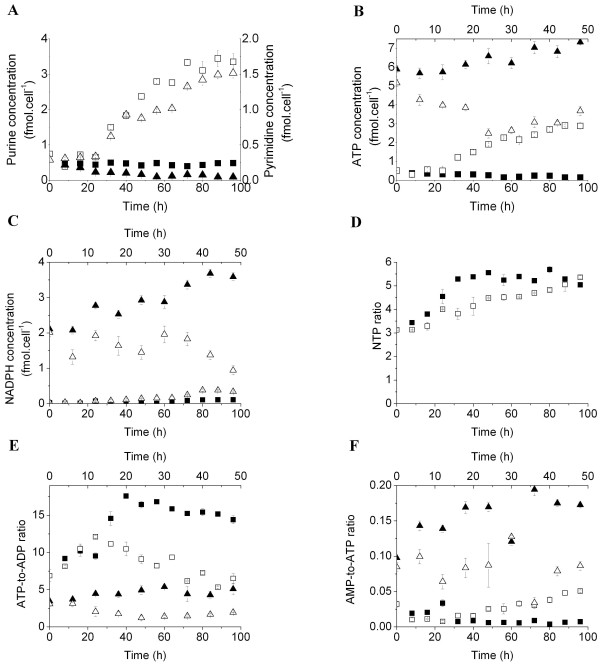
** Bioenergetic status of BM-derived MDSCs.** (**A**) Specific cell concentration in purines (GTP + ATP + ADP + AMP, squares) and pyrimidines (CTP + UTP + UDPGNAc, triangles). (**B**) Specific cell concentration of ATP. (**C**) Specific cell concentration of NADPH. Nucleotide-derived behavioral markers: (**D**) NTP ratio (ATP + GTP)/(UTP + CTP) in BM cells and BM-derived MDSC cultures. (**E**) ATP-to-ADP ratio. (**F**) AMP-to-ATP ratio.

Consistent with the stimulation of central carbon metabolism during the maturation process, the BM-derived MDSC bioenergetic state was up-regulated. The intracellular pools of purines (GTP + ATP + ADP + AMP), which represent energy-related nucleotides, and pyrimidines (CTP + UTP + UDPGNAc), which are involved in various anabolic reactions and growth-related metabolic processes [16,17], increased at the respective rates of 0.030 ± 0.007 and 0.014 ± 0.002 fmol/cell/h after 24 h of treatment (Figure 4A). The BM-derived MDSC energetic pool mainly constituted of purines (60 to 70%), an expected result considering the low specific growth rate. Conversely, the purine pool was relatively stable at 0.46 ± 0.03 fmol/cell/h in the control culture, and pyrimidine levels showed a decrease due to cell quiescence (Figure 4A). In particular, ATP was accumulated at a rate of 26.398 ± 2.789 × 10^-3^ fmol/cell/h from 24 h. The enhanced activity of the TCA cycle may thus have indirectly contributed to the production of ATP, since glycolytic metabolism has a low energy yield (Figure 4B). Furthermore, the cell specific concentration of NADPH increased at a rate of 1.68 ± 0.04 × 10^-3^ fmol/cell/h between 24 and 64 h (Figure 4C). The rate of NADPH accumulation shifted rapidly to 9.72 ± 0.02 × 10^-3^ fmol/cell/h between 64 and 80 h. In addition to the role of the oxidative phase of PPP in NADPH production, malate dehydrogenase and isocitrate dehydrogenase produce considerable amounts of NADPH along with pyruvate and α-ketoglutarate, respectively. Thus, ATP and NADPH production rates were higher than the demand for maturation and the resulting immunosuppressive machinery. The cell specific NADPH concentration slightly decreased from 0.382 ± 0.031 fmol/cell at 80 h to 0.0341 ± 0.016 fmol/cell at the end of the culture (96 h), suggesting either higher demands from NADPH-requiring processes or the down-regulation of NADPH production.

The inhibition of iNOS and ARG1 activities decreased the cell specific ATP concentration from 5.16 ± 0.20 fmol/cell (at inoculation) to 2.49 ± 0.25 fmol/cell after 24 h, and then increased at a rate of 0.046 ± 0.007 fmol/cell/h until the end of the culture (96 h) (Figure 4B). However, in the control culture, ATP continuously accumulated at a rate of 0.033 ± 0.002 fmol/cell/h to a maximal value of 7.33 ± 0.12 fmol/cell/h at 48 h. Similarly, MSC-1 cell specific NADPH concentrations were stable at 1.60 ± 0.03 fmol/cell in the presence of 1400 W and BEC, whereas it progressively accumulated at a rate of 0.034 ± 0.009 fmol/cell/h in the control culture (Figure 4C).

An exhaustive study of nucleotide-derived biomarkers showed that cell metabolic activity increased as MDSC maturation progressed. Treating BM cells with GM-CSF and IL-6 increased the NTP ratio, defined as ([ATP] + [GTP])/([UTP] + [CTP]) the ratio of energetic nucleotides to anabolism-related nucleotides [17], from 3.14 ± 0.07 (during the first 24 h) to 5.36 ± 0.06 at the end of the culture (96 h) (Figure 4D). An increase in the NTP ratio normally indicates the deterioration of cell metabolic activity if the cell growth process was active. In this case, the rates of ATP (Figure 4B) and GTP (data not shown) accumulation were higher than those of CTP and UTP (data not shown), indicating that BM-derived MDSCs were catabolically active. Nevertheless, the NTP ratio of the control culture increased rapidly after cell inoculation, and reached a plateau (5.34 ± 0.014) at 32 h. This increase was mostly caused by a decrease in pyrimidines in the cell pool.

Although the cell specific ATP concentration increased during culture (Figure 4B), ATP was continuously depleted from the intracellular pool during maturation as the ATP-to-ADP ratio, a marker of respiration and energy consumption, decreased [18] (Figure 4E). Indeed, the ATP-to-ADP ratio in the BM-derived MDSC culture was 2 to 3 times lower than that of the control culture, and decreased at a specific rate of 0.058 ± 0.002 per h. However, the ATP-to-ADP ratio in iNOS and ARG1-inhibited MSC-1 cell cultures decreased from 3.08 ± 0.38 (at inoculation) to 1.22 ± 0.31 after 24 h, with a slight increase thereafter (Figure 4E). Conversely, this ratio continuously increased from 3.46 ± 0.19 (at inoculation) to a plateau value of 4.82 ± 0.38 at 30 h in the control culture. This decrease was probably caused by anabolic-related energy consumption, since inhibitors did not induce any remarkable effects on cell growth or viability.

However, the AMP-to-ATP ratio, which is considered a marker of glycolysis stimulation and a regulator of AMPK activity [19], shifted after 24 h and was considerably higher than in the control culture, indicating sustained ATP production (Figure 4F). Moreover, the AMP-to-ATP ratio in the iNOS and ARG1-inhibited MSC-1 cell culture was 2 to 5-fold lower than that of the control culture, suggesting AMPK activity is probably regulated by the cellular immunosuppressive potential (Figure 4F).

### Role of AMPK in BM-derived MDSC maturation

**Figure 5:**
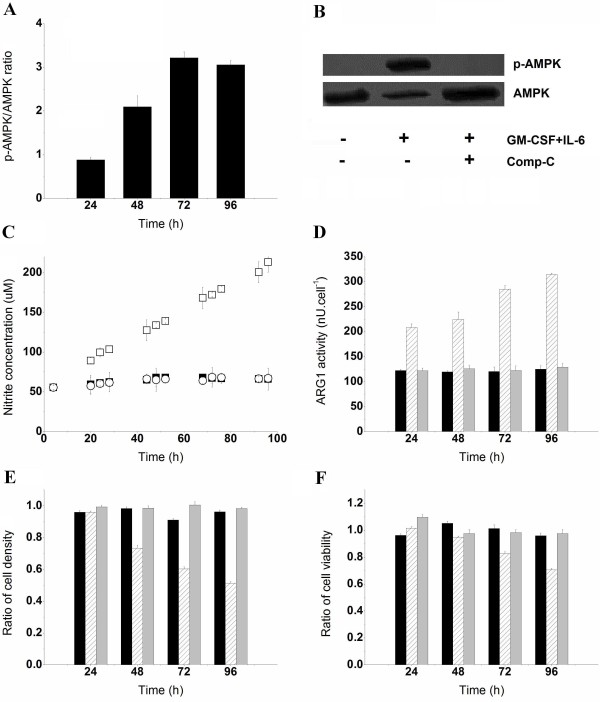
** Implication of AMPK in the maturation of BM cells to MDSCs.** (**A**) p-AMPK to AMPK ratio in BM-derived MDSCs as analyzed by densitometry analysis of western blot bands. (**B**) p-AMPK and AMPK after 96 h in BM cells, BM-derived MDSCs, Comp-C pre-treated BM cells (5 μM, 15 min) cultured in the presence of GM-CSF and IL-6 (40 ng/ml). (**C**) Nitrate and nitrite concentrations in supernatants. Filled and empty squares (■, □) correspond to BM cells and BM-derived MDSC cultures, respectively. Empty circles (○) represent Comp-C pre-treated BM cells cultured in the presence of GM-CSF and IL-6. (**D**) ARG1 activity. Untreated BM cells (Black), BM cells exposed to GM-CSF and IL-6 for 24, 48, 72 or 96 h (hatched) and Comp-C pre-treated BM cells rinsed with sterile PBS and then cultured in the presence of GM-CSF and IL-6 (Grey). The same nomenclature is used for Figure 5E and F. (**E, F**) Ratios (referenced to the control culture) of Jurkat cell density and viability respectively.

The increase of AMP-to-ATP ratios observed during the MDSC maturation process suggested that AMPK is implicated in the regulation of cells bioenergetics. This hypothesis was confirmed by western blot analysis, where the expression level of phosphorylated AMPK (p-AMPK) increased in the presence of GM-CSF and IL-6 (Figure 5A), whereas p-AMPK was not detectable in the BM cell culture (Figure 5B). Moreover, AMPK is critical for prostaglandin E2 (PGE2)-induced differentiation of BM cells to endothelial progenitor cells [20,21], and PGE2-induced differentiation of CD11b^+^/Gr-1^+^ MDSCs in tumor-bearing animals [22]. We have then verified whether AMPK inhibition interferes with the acquisition of BM-derived MDSCs immunosuppressive potential. Treating BM cells with 5 μM of Comp-C for 15 min was sufficient to inhibit the phosphorylation of AMPKα1/2 at Thr172 throughout the 96 h culture (Figure 5B), without affecting AMPK expression levels or cell growth and viability (data not shown). As expected, the inhibition of AMPK activity by Comp-C resulted in a lower glucose uptake rate of 0.157 ± 0.011 pmol/cell/h, when compared to the GM-CSF and IL-6–treated BM cell culture (0.293 ± 0.007 pmol/cell/h). Pre-treatment of BM cells with Comp-C prior to cytokine addition maintained the lactate-to-glucose ratio at a lower value (0.88 ± 0.04) compared with the high lactate-to-glucose yield (1.82 ± 0.03) observed in the BM-derived MDSC culture. This suggests that AMPK-inhibited BM cells cultured in the presence of GM-CSF and IL-6 favored energy production rather than lactate accumulation, or that the L-Gln-derived lactate production rate had decreased. However, L-Gln did not compensate for the decrease of glucose-derived carbon, since the L-Gln uptake rate slightly decreased in the presence of Comp-C (0.017 ± 0.003 pmol/cell/h) when compared with the control culture (0.023 ± 0.004 pmol/cell/h). In addition to lower nutrient uptake rates, the inhibition of AMPK activity was accompanied by inhibition of iNOS and ARG1 activity. The concentration of nitrate and nitrite, markers of iNOS activity, remained constant at 63.80 ± 3.41 μM in AMPK-inhibited BM cells cultured with GM-CSF and IL-6, whereas BM-derived MDSCs produced nitrate and nitrite at a rate of 8.13 ± 0.13 fmol/cell/h (Figure 5C). Similarly, GM-CSF and IL-6 failed to activate ARG1 when AMPK was inhibited since ARG1 activity remained constantly low at (124.63 ± 2.64 nU/cell) similar to the control culture (Figure 5D). ARG1 activity increased at different rates and reached high values (314.30 ± 2.63 nU/cell) in the BM-derived MDSC culture. The inhibition of iNOS and ARG1 activities in the AMPK-inhibited BM cells abolished the immunosuppressive potential since cells failed to inhibit the growth and viability of Jurkat cells. However, BM-derived MDSCs reduced Jurkat cell density and viability by 49.9 ± 3.71 and 27.1 ± 1.1%, respectively (Figure 5E, F).

## Discussion

Co-induction of ARG1 and iNOS by bacterial lipopolysaccharide in macrophages modulates central carbon metabolism and respiration [23,24], and the cell bioenergetic state. Since tumor-infiltrating MDSCs have the highest immunosuppressive potential among different MDSC sub-populations [6], we simulated the maturation of BM precursors to MDSCs using a combination of GM-CSF and IL-6, in vitro. Complementary to work conducted by Marigo et al., where BM-derived MDSCs were harvested after 96 h of treatment [5], we continuously monitored the progression of MDSC maturation every 8 h. We also characterized MSC-1 cells nutritional profile and energetic states when iNOS and ARG1 activities were inhibited, to analyze the immunosuppression-related energy demand. A greater understanding of immunosuppression at the metabolic level may be significant for the identification of new immunotherapy targets.

Exposure of BM cells to GM-CSF and IL-6 induced a continuous up-regulation of iNOS and ARG1 activities after 24 and 16 h, respectively (Figure 1A1B). The delayed effects can be attributed to cytokine internalization and to activation of the CCAAT-enhancer-binding protein (C/EBP)β transcription factor [5] and other signaling pathways (JAK/STAT3, MAPK and PI3-K) that were shown to regulate the expression and activation of L-Arg metabolizing enzymes [25]. However, BM-derived MDSCs only exhibited their immunosuppressive potential after 48 to 72 h exposure to cytokines, which corresponded to the initiation of Jurkat cell growth inhibition and loss of viability, respectively (Figure 1C, D). The immunosuppressive activity of BM-derived MDSCs was not observed until sufficient L-Arg was sufficiently removed from the culture medium allowing NO derivatives to accumulate.

The activation of iNOS and ARG1 was accompanied by the up-regulation of glucose uptake (Figure 2A) and glycolysis, as shown by the AMP-to-ATP ratio, which was up to 5-fold higher than in the control culture (Figure 4F), and by the accumulation of G-6-P and F-6-P (Figure 2B). This accumulation may suggest that cells continue producing these intermediates without consuming them, a behavior previously associated with the initiation of cell death [17]. However, cells remained viable and grew throughout the culture. L-Arg and lactate were continuously produced, suggesting that cells consumed these intermediates to support both the catabolic processes and the sparse synthesis of anabolism-related macromolecules. Thus, the accumulation is caused by a higher rate of production compared with consumption. The cell specific concentration of F-6-P, which was approximately 60% to 70% that of G-6-P, suggests that glucose is mainly processed by glycolysis (Figure 2B). This may be a consequence of the low specific growth rate exhibited by BM-derived MDSCs, which may have limited fluxes of the non-oxidative reactions of the PPP. These results are in agreement with previous studies by Ando et al., where IL-6 enhanced the expression of the glycolytic enzymes hexokinase 2 and 6-phosphofructo-2-kinase/fructose-2,6-biphosphatase-3 in mouse embryonic fibroblasts via the IL-6/STAT3 pathway [26]. Similarly, GM-CSF promoted rapid glucose transport in *Xenopus* oocytes via the PI3-K pathway [27]. L-Gln uptake was also increased in the presence of GM-CSF and IL-6 (Figure 3A). In addition to the possible direct effect of cytokines on nutrient uptake, the enzymatic activities of iNOS and ARG1 indirectly regulated glucose and L-Gln consumption since the inhibition of iNOS and ARG1 activities in MSC-1 cells down-regulated glucose and L-Gln uptake. Therefore, the abolition of MSC-1 cells immunosuppressive potential in the presence of 1400 W and BEC may have decreased cellular demand of intermediates and energy, and so regulated nutrient uptake to adjust to the lower requirements. The decrease of nutrient uptake was not caused by the direct effects of 1400 W and/or BEC on the expression or activity of glucose or glutamine transporters. The presence of these inhibitors in Jurkat cell cultures did not induce noticeable effects at the nutritional level (data not shown), although Jurkat cells and macrophages, as principal sub-populations of MSC-1 cells, express the same nutrient transporters [28,29]. This hypothesis requires further confirmation using specific siRNA to iNOS and ARG1 to avoid possible unknown effects associated with inhibitors that may have caused the down-regulation of nutrient uptake.

Stimulation of glycolysis and glutaminolysis provides the TCA cycle with specific intermediates, thus ensuring its enhanced activity (Figure 3B, C, D). Interestingly, malate accumulation was initiated around 8 to 16 h before fumarate and α-KG accumulation. This may appear illogical since fumarate and α-KG are the upstream metabolites of malate. The study of metabolic fluxes and enzymatic kinetics is therefore crucial for understanding the differences in the trends of metabolite concentrations. The use of labeled nutrients may also offer interesting insight to the origin and fate of each metabolite. Particularly, the accumulation of fumarate suggests that L-Arg is continuously synthesized and supports a permanent immunosuppressive activity (Figures 1,3C). Furthermore, the enhanced activity of the TCA cycle resulted in the accumulation of high levels of malate compared to PEP (Figures 2D3D), both of which are precursors of pyruvate. This may suggest that the TCA cycle contributes to lactate accumulation since the lactate production-to-glucose consumption rates reached values higher than 2 (Figure 2D). BM-derived MDSCs undergo anaerobic glycolysis despite non-limiting oxygen conditions, a typical behavior for tumor cells that produce lactate rather than obtaining high energy yields from respiration. This phenomenon results in the acidification of the tumoral microenvironment, a condition that promotes tumor progression and metastasis [30]. Moreover, the accumulation of fumarate was previously associated with the inhibition of HIF hydroxylases and the stable expression of HIF [31]. On that note, our results agree with recent findings demonstrating that MDSCs express hypoxia-induction factor-1 α to adapt to the quasi-hypoxic conditions encountered in tumors [32]. Similarly, previous work by Wada et al. revealed that GM-CSF induces a rapid glucose-dependent extracellular acidification that is regulated by protein kinase C and the sodium/proton antiporter [33]. Moreover, iNOS activity is associated with an increased glucose consumption rate, increased glycolysis and PPP, and the inhibition of oxidative phosphorylation in zymosan-treated macrophages [34]. Likewise, a study by Irace et al., on LPS-treated macrophages reported a bi-directional regulation between NO and TCA cycle, that supports our findings. NO was shown to regulate aconitase activity and α-ketoglutarate production and alterations of the TCA cycle correlated with the inhibition of NO biosynthesis [35].

The two principal NADPH producing pathways in mammalian cells, the TCA cycle and PPP, were also stimulated in the presence of GM-CSF and IL-6. As the non-oxidative phase of PPP is devoted to anabolic processes and BM-derived MDSCs exhibit a low growth rate, the conversion of G-6-P into F-6-P was probably dominant to the production of 6-phosphogluconolactone, the first PPP intermediate. However, NADPH is mostly derived from the TCA cycle, particularly via malate dehydrogenase and isocitrate dehydrogenase, since fluxes through these pathways were considerably higher than those through the PPP (Figures 2C, 3B–D), although the oxidative phase of the latter was shown to be stimulated. As BM-derived MDSCs exhibited low specific growth rates in vitro, the oxidative phase, which is responsible for NADPH production, was probably more active than the non-oxidative phase, which is related to anabolic processes. This resulted in the recycling of PPP intermediates to the glycolysis pathway.

Although BM-derived MDSCs undergo glycolytic metabolism, with a low energy yield, the specific cell concentration of ATP increased gradually during maturation (Figure 4B). However, the decreasing trend of the ATP-to-ADP ratio suggests that ATP was continuously depleted from the intracellular pool (Figure 4E). This may be due to ATP consumption, forming ADP, or ADP production from AMP. Nevertheless, the production of ADP from AMP, via the enzymatic activity of adenylate kinase, requires ATP. Thus, the decrease of the ATP-to-ADP ratio was probably caused by ATP consumption.

Enhanced AMPK activity, as suggested by the continuous increase of the AMP-to-ATP ratio (Figure 4D) and confirmed by p-AMPK blotting (Figure 5A), is most likely responsible for the up-regulation of ATP-producing processes. Indeed, AMPK is considered an energy sensor in several metabolic disorders, such as cancer and diabetes, where enzymes switch cellular metabolism from anabolic to catabolic in reaction to deficits in cellular energy [36]. IL-6 up-regulated AMPK activity in rat skeletal muscle cells, and the IL-6-induced STAT3 enzyme was localized in mitochondria, resulting in enhanced oxidative phosphorylation and consequently increased cell ATP levels [37,38]. However, we observed that BM-derived MDSC respiration decreased in the presence of GM-CSF and IL-6, probably caused by HIF-1α expression [32]. The role of AMPK in HIF-1α expression is cell type dependent. Hypoxia induces HIF-1α expression in an AMPK-independent manner in mouse embryonic fibroblasts, whereas enhanced AMPK activity is important for HIF-1α transcriptional activity under hypoxic conditions in prostate cancer cell lines [39]. Therefore, the enhanced activity of AMPK in BM-derived MDSCs may have had multiple effects, by switching cells from oxidative phosphorylation to glycolysis, supported by Cidad et al., where inhibition of mitochondrial respiration by NO rapidly stimulated glucose uptake through AMPK [19]. Alternatively, to compensate for a deficient energy yield from glycolytic metabolism, BM-derived MDSCs further stimulated TCA cycle activity through glutaminolysis to produce ATP at a higher rate than required by maturation and other biochemical reactions. Nevertheless, the relative low ATP-to-ADP ratio in the iNOS and 1400 W-inhibited MSC-1 cell culture indicated that the ATP consumption rate was higher than that of its net production, when compared to the control culture. However, this low ATP production rate did not stimulate AMPK in response to the energy deficit as revealed by the low AMP-to-ATP ratio when compared to the control culture. This suggests that the enzymatic activities of iNOS and ARG1 probably regulate AMPK activity, and that their inhibition may render AMPK unresponsive to energy deficit.

To confirm the implications of AMPK in the maturation process of BM cells to MDSCs, we incubated freshly isolated BM cells with Comp-C, a potent selective and ATP-competitive inhibitor of AMPK [40]. Since Comp-C is a reversible inhibitor, the expression levels of p-AMPK were monitored throughout the duration of culture to confirm the inhibition of AMPK activity (Figure 5B). The inhibition of AMPK in Comp-C pre-treated BM cells cultured in the presence of GM-CSF and IL-6 caused a net decrease in the uptake rate of major nutrients (glucose and L-Gln). This may have resulted in the down-regulation of TCA cycle activity and of the related metabolic pathways, such as L-Arg endogenous synthesis and energy production. Furthermore, AMPK inhibition down-regulated the GM-CSF and IL-6–induced activation of iNOS and ARG1 (Figure 5C, D). NADPH, a co-factor of iNOS, is principally derived from the glutaminolysis/TCA cycle axis and the decrease of cell specific levels of NADPH caused the down-regulation of iNOS activity. This finding is consistent with a previous report where the inhibition of AMPK activity by Comp-C, or dominant negative AMPK, down-regulated the activity of PGE2-induced eNOS (endothelial isoform of NOS), in human epithelial progenitor cells [20]. Interestingly, GM-CSF and IL-6 failed to activate ARG1 in the AMPK-inhibited BM cells (Figure 5D), although we recently showed that ARG1 activity was not associated with any specific energy requirements [41]. The activation of the mitogen-activated protein kinase (MAPK) signaling pathways including p38MAPK, ERK1/2 and SAPK/JNK, which are implicated in BM-derived MDSC maturation [5], was strictly linked to the induction of iNOS expression in macrophages [42]. Moreover, the AMPK inhibition-induced down-regulation of L-Arg endogenous synthesis and the low affinity of ARG1 for L-Arg (approximately 10 mM) may have contributed to the inactivation of ARG1. The inhibition of AMPK activity in macrophages was also shown to inhibit cyclooxygenase-2 activity, which is crucial for MDSC accumulation in the tumor microenvironment [43,44]. Therefore, AMPK, via downstream-activated signaling pathways, is implicated in the maturation of BM cells to MDSCs. Although the phenotype of GM-CSF and IL-6-treated AMPK-inhibited BM cells was not investigated, iNOS and ARG1 activities were suppressed and led to a non-immunosuppressive cell population, as revealed by cytotoxicity assay, where the density and viability of Jurkat cells cultured in the presence of cytokine-treated Comp-C-pre-treated BM-cells were similar to those observed in the control culture (Jurkat cells only) (Figure 5E, F). As for all pharmacological inhibitors, Comp-C may have off-target effects that affect the reliability of the results observed here. Although no Comp-C-related side effects on cell metabolism were reported in the literature, Comp-C could potentially interact with other cell compartments, proteins or other molecules resulting in experimental bias. Studying the maturation of BM cells to MDSCs when the AMPK gene is silenced, using either AMPK knockout mice or specific siRNA to AMPK, is thus crucial before initiating *in vivo* studies.

Both experimental models suggest that AMPK, iNOS and ARG1 are co-activated. Several hypotheses can be suggested to explain the concomitant activation of iNOS, ARG1 and AMPK and the up-regulation of central carbon metabolism. First, all these enzymes and pathways may have been up-regulated by a direct effect of GM-CSF and IL-6. Second, the activation of iNOS and ARG1 may have increased cells demand for energy. AMPK may have been activated to respond to the energy deficit since BM-derived MDSCs and MSC-1 cells exhibit glycolytic metabolism. However, the regulatory mechanisms between AMPK, iNOS and ARG1 require further investigation to determine signaling pathways and signals implicated in this bi-directional regulation.

## Conclusions

In conclusion, the present study suggests that alterations of myelopoiesis, and thus MDSC maturation and the consequent activation of iNOS and ARG1, may depend on enhanced central carbon metabolism and up-regulation of the cell bioenergetic state. Furthermore, our results for nucleotide-derived behavioral biomarkers, and on the distribution of TCA cycle intermediates, unambiguously indicate that AMPK is involved in the GM-CSF and IL-6–induced stimulation of glycolysis and TCA cycle. Moreover, mature MDSCs, as for its immortalized form (MSC-1 cells), undergo anaerobic glycolysis and partially oxidize L-Gln to ensure favorable conditions for tumor progression, as do cancer cells. These results may thus have clinical relevance, since the modulation of metabolic fluxes through glycolysis and glutaminolysis, via the inhibition of AMPK activity, may efficiently impair MDSC maturation and their immunosuppressive activity and indirectly help to recover anti-tumoral immune responses.

## Methods

### Mice

Six- to eight-week-old male C57BL/6 mice were purchased from Charles River (Quebec, Canada) and maintained under specific pathogen-free conditions in the animal facilities of the Université de Montréal. Experiments were performed according to state guidelines and approved by the Canadian Council on Animal Care.

### BM cell culture

Single cell suspensions were prepared from BM of normal mice and cultured in 100-mm Petri dishes (Becton Dickinson, Quebec, Canada) in 10 ml of RPMI 1640 medium (Sigma, Ontario, Canada) supplemented with 10% (v/v) irradiated fetal bovine serum (Cedarlane, Ontario, Canada), 1 mM Sodium Pyruvate (Sigma), 50 μM β-mercaptoethanol (Sigma), 100 U/ml Penicillin, 150 U/ml Streptomycin (Cedarlane) and 2 mM L-glutamine (Cedarlane), in a 5% CO_2_ and 37 °C incubator. MDSCs were derived by treating BM cells with 40 ng/ml of GM-CSF and 40 ng/ml of IL-6 (both from Cedarlane) for 4 days as described by Marigo et al. [5].

When required, BM cells were treated for 15 min with 5 μM of Compound C (Comp-C, 6-[4-(2-Piperidin-1-yl-ethoxy)-phenyl)]-3-pyridin-4-yl-pyrrazolo[1,5-a]-pyrimidine, Sigma) dissolved in DMSO (1% v/v) to inhibit AMPK activity.

Prior to analysis, cells were detached using a phosphate buffer saline (PBS)-EDTA (2 mM) solution, rinsed with PBS and centrifuged for 6 min at 250 × *g* at 4 °C. Cells were counted using a hematocytometer and cell viability was determined by the Trypan Blue exclusion method.

### MSC-1 cell culture

The generation, culture, and phenotype of the MSC-1 immortalized cell line has been described previously [8]. MSC-1 cells were grown in 75 cm^2^ T-flasks (VWR, Ontario, Canada) in RPMI 1640 medium (Sigma) supplemented with 10% (v/v) irradiated FBS (Cedarlane), 1 mM sodium pyruvate (Sigma), 100 U/ml Penicillin, 150 U/ml streptomycin (Cedarlane) and 2 mM L-glutamine (Cedarlane), in a 5% CO_2_ and 37 °C incubator. Cultures were inoculated at a cell density of 0.2 × 10^6^ cells/ml and cells were passaged when they reached 80% confluence.

When required, iNOS and ARG1 activities were inhibited with 100 μM of 1400 W and 5 μM of BEC (both from Cedarlane), respectively.

Prior to analysis, cells were detached using 0.25% Trypsin and 1 mM EDTA (Invitrogen, Ontario, Canada), rinsed with PBS and centrifuged for 6 min at 250 × *g* at 4 °C. Cells were counted using a hemacytometer and viability was determined using the Trypan Blue exclusion method.

### Assays

Glucose, lactate, glutamate and glutamine concentrations in supernatants were measured using a dual-channel immobilized oxidase enzyme biochemistry analyzer (2700 SELECT, YSI Inc. Life Sciences, Yellow Springs, OH, USA), using calibration buffers provided by the manufacturer.

Nitric oxide concentrations in supernatant were respectively assayed using a Nitrate/Nitrite Colorimetric Assay Kit (Cedarlane). Specific rates of nutrients consumption and metabolites production were calculated using the average method [45].

### SDS-PAGE and western blot analyses

Detached cells were lysed in 100 μl of lysis buffer (50 mM HEPES, pH 7.4, 150 mM NaCl, 1% thesit, and 0.5% sodium deoxycholate), and insoluble material was removed by centrifugation at 10,000 × *g* for 5 min at 4°C. Samples were mixed 3:1 with sample buffer containing 0.6 mM DTT and boiled for 5 min. Proteins were separated by 10% SDS-PAGE at 200 V during 45 min. For Western blots, proteins were transferred to a PVDF membrane (Bio-Rad, Mississauga, Ontario, Canada) using Tris-glycine buffer for 1 h at 200 mA. The membrane was blocked with 5% skim milk/10 mM Tris–HCl, pH7.4, 150 mM NaCl, 0.1% Tween-20 and then probed with the appropriate primary antibodies (diluted 1:200) for AMPK α1/2 and p-AMPK α1/2 (Thr172) (Santa Cruz Biotechnology, Santa Cruz, CA) overnight at 4 °C. Specific antibody binding was detected using goat anti-rabbit IgG horseradish peroxidase (diluted 1:1000, R&D Systems, Minneapolis, MN) for 1 h at room temperature and visualized using an enhanced chemiluminescence detection reagent (Bio-Rad). Band intensities were compared with ImageJ software.

### Determination of ARG 1 activity

Total cells were lysed with 50 μl of a lysis buffer containing 0.1% Triton X-100 (Sigma) and 100 μg/ml of pepstatin, antipain and aprotinin (all from EMD BioSciences, San Diego, CA). After 30 min in a thermomixer at 37 °C, cell debris was removed by centrifugation at 15,000 × *g* for 20 min and cell lysates were kept at −80 °C prior to analysis. ARG1 activity was quantified in cell lysates by urea determination with α-isonitrosopropiophenone as previously described by Munder et al., [46]. One unit of ARG1 activity is defined as the enzyme activity that catalyzes the production of 1 mol urea/min.

### Cytotoxicity assay

The immunosuppressive activity of BM-derived MDSCs and MSC-1 cells was assessed by their ability to inhibit Jurkat cell growth (leukemic T-cells, clone E6-1, Cedarlane). Experiments were performed in 24-well tissue culture plates (VWR, Ontario, Canada) in a final volume of 1 ml. Jurkat cells were inoculated (500 μl at 0.2 × 10^6^ cells/ml) in Millicell®PC 0.4 μm culture plate inserts (Millipore) and added to wells containing 500 μl of BM cell suspension (0.2 × 10^6^ cells/ml) cultured in the presence of GM-CSF and IL-6 (40 ng/ml each) for 0, 24, 48, 72 and 96 h or to MSC-1 cells cultured in the presence of 1400 W and BEC (100 and 5 μM, respectively) for 16 h (500 μl at 0.2 × 10^6^cells/ml).

To investigate the role of AMPK in the maintenance of BM-derived MDSCs immunosuppressive potential, Comp-C pre-treated BM cells cultured in the presence of GM-CSF and IL-6 (for 24, 48, 72 and 96 h) were rinsed with sterile PBS to remove Comp-C and re-suspended in GM-CSF and IL-6 (40 ng/ml each) enriched complemented culture medium. Mixed cultures were kept in a 5% CO_2_ and 37 °C incubator for 24 h for BM cells and BM-derived MDSCs, and for 32 h for MSC-1 cells. Jurkat cells were then counted using a hemacytometer and viability was determined using the Trypan Blue exclusion method. The small pore size of the culture inserts prevented Jurkat cells and detached BM-derived MDSCs and MSC-1 cells to cross-contaminate respective media/cell samples and so ensured the reliability of cell counts.

### Respirometry test

Respirometry assays were performed as described by Lamboursain et al., [47]. Briefly, 3 ml of a 15 × 10^6^ BM-derived MDSCs/ml suspension or 3 ml of a 5 × 10^6^ MSC-1 cells/ml suspension were inoculated in a 10-ml borosilicate glass syringe (Sigma) in which the plunger was substituted by an Ingold pO_2_ probe (Mettler Toledo, Quebec, Canada). The respirometer was kept at 37 °C and magnetically agitated (60 RPM) to ensure the homogeneity of cell suspension. Dissolved oxygen was recorded by an acquisition system (Centris, Longueuil, Canada).

### Metabolite extraction

The extraction protocol was based on the method developed by Kimball et al., [48]. Briefly, for each sample, 5 × 10^6^ cells were extracted with 400 μl of 80% cold methanol in the presence of 0.2 g of sand (Sigma). After 10 min on dry ice, the mixture was vortexed and then sonicated in ice and water for 5 min. The samples were then centrifuged for 7 min at 21,000 × *g* and 4 °C to collect supernatants. The pellets were extracted a second and third time as described above with 200 μl of 50% cold methanol and 200 μl of cold water, respectively. Supernatants were mixed and stored at −80 °C prior to analysis. Extracts were filtered through 0.2 μm filters (Millipore, Ontario, Canada) before analysis.

### Nucleotide concentrations

Nucleotides in BM cell and BM-derived MDSC extracts were analyzed using a 1290 UPLC system coupled to a 6460 triple quadruple mass spectrometer (both from Agilent Technologies, Quebec, Canada). Nucleotides were separated by a Symmetry C18 column (150*2.1 mm, 3.5 μm) (Waters) equipped with a Security C18 guard-column (Waters, 10*2.1 mm, 3.5 μm) by the ion-pair method. DMHA (N,N-dimethylhexylanine, Sigma) was used as an ion-pair reagent to improve the signal-to-noise ratio with positive ionization mode. The mobile phase consisted of Buffer A: 10 mM ammonium acetate, 15 mM DMHA at pH 7.0, and Buffer B: 50/50% (v/v) acetonitrile, 20 mM NH_4_OAc at pH 7.0. Mobile phase flow rate was set at 0.3 m/min with the following gradient: 0–10 min at 10% B, 10–20 min at linear gradient from 10 to 30% B, 20–21 min at linear gradient from 30 to 60% B, 21–26 min at 60% B, 26–27 min at linear gradient from 60 to 10% B and 27–35 min at 10% B.

Nucleotide concentrations in MSC-1 cell extracts were determined by ion-pairing liquid chromatography-electrospray ionization mass spectrometry (positive mode) using a HPLC-MS system (Waters, Milford, MA) equipped with a Symmetry C18 column (150*2.1 mm, 3.5 μm) (Waters) and a Security C18 guard-column (Waters, 10*2.1 mm, 3.5 μm). Likewise, DMHA was used as an ion-pair reagent. The mobile phase consisted of Buffer A, 10 mM ammonium acetate, 15 mM DMHA at pH 7.0, and Buffer B, 40% (v/v) acetonitrile in water. The flow rate was set at 0.3 ml/min using the following gradient: 0–10 min at 15% B, 10–12 min at linear gradient from 15 to 40% B, 12–30 min at linear gradient from 40 to 70% B, 30–35 min at 70% B, 35–37 min at linear gradient from 70% to 15% B and 37–45 min at 15% B. In both cases, quantification of metabolites (nucleotides and organic acids) was performed by integrating peak areas and using calibration curves. Cell specific concentrations in metabolites were calculated by normalizing the quantity of metabolites in cell extracts to the number of extracted cells.

### Organic acid concentrations

Organic acids concentrations were assessed using the above-mentioned UPLC-MS/MS system using a Hypercarb column (100*2.1 mm, 5 μm) and a Hypercarb pre-column (2.1*10, 5 μm) (Thermo Fisher, Ontario, Canada). The mobile phase consisted of Buffer A, 20 mM ammonium acetate at pH 7.5, and Buffer B, 10% (v/v) methanol in water. The flow rate was set at 0.3 ml/min using the following gradient: 0–5 min at 10% A, 5–10 min at linear gradient from 10% to 20% A, 10–20 min at linear gradient from 20% to 100% A, 20–30 min at 100% A, 30–32 min at linear gradient from 100% to 10% A and 32–40 min at 10% A.

### Statistical analysis

Data are shown as mean ± SEM (standard error of mean) of *n* = 3 independent experiments from 3 distinct cell cultures.

## Abbreviations

α-KG, α-ketoglutarate; AMPK, AMP protein kinase; ARG1, Arginase 1; BM, Bone marrow; Comp-C, Compound C; F-6-P, Fructose-6-phosphate; G-6-P, Glucose-6-phosphate; GM-CSF, Granulocyte macrophage – colony stimulating factor; IL-6, Interleukin-6; iNOS, Inducible nitric oxide synthase; L-Arg, L-arginine; L-Gln, L-glutamine; MDSCs, Myeloid-derived suppressor cells; MSC-1, Myeloid suppressor cells-1; NO, Nitric oxide; PEP, Phosphoenolpyruvate; PPP, Pentose phosphate pathway; TCA, Tricarboxylic acid; TDSFs, Tumor-derived soluble factors.

## Competing interests

The authors declare that they have no competing interests.

## Authors’ contributions

IH designed the study with MJ. IH conceived the methods, performed cell culture and cell extraction, analyzed and interpreted the data and wrote the manuscript. JC participated in metabolite analysis. FM assisted in SDS PAGE and Western blot analyses. MJ, VB and GDC supervised the study. All authors revised, read and approved the final manuscript.
